# Expanding the Junction: New Insights into Non-Occluding Roles for Septate Junction Proteins during Development

**DOI:** 10.3390/jdb9010011

**Published:** 2021-03-21

**Authors:** Clinton Rice, Oindrila De, Haifa Alhadyian, Sonia Hall, Robert E. Ward

**Affiliations:** 1 Department of Molecular Biosciences, University of Kansas, Lawrence, KS 66045, USA; clinton.rice11@gmail.com (C.R.); haifaalhadyian@ku.edu (H.A.); 2 Department of Biology, Case Western Reserve University, Cleveland, OH 44106, USA; oxd47@case.edu; 3 BioKansas, Shawnee, KS 66217, USA; Sonia@biokansas.org

**Keywords:** septate junction, epithelia, *Drosophila*, morphogenesis, apical/basal polarity, planar polarity, wound healing, dorsal closure, organogenesis, secretion

## Abstract

The septate junction (SJ) provides an occluding function for epithelial tissues in invertebrate organisms. This ability to seal the paracellular route between cells allows internal tissues to create unique compartments for organ function and endows the epidermis with a barrier function to restrict the passage of pathogens. Over the past twenty-five years, numerous investigators have identified more than 30 proteins that are required for the formation or maintenance of the SJs in *Drosophila melanogaster*, and have determined many of the steps involved in the biogenesis of the junction. Along the way, it has become clear that SJ proteins are also required for a number of developmental events that occur throughout the life of the organism. Many of these developmental events occur prior to the formation of the occluding junction, suggesting that SJ proteins possess non-occluding functions. In this review, we will describe the composition of SJs, taking note of which proteins are core components of the junction versus resident or accessory proteins, and the steps involved in the biogenesis of the junction. We will then elaborate on the functions that core SJ proteins likely play outside of their role in forming the occluding junction and describe studies that provide some cell biological perspectives that are beginning to provide mechanistic understanding of how these proteins function in developmental contexts.

## 1. Introduction

The septate junction (SJ) serves as the occluding junction in epithelial tissues in invertebrate organisms [1]. Functionally similar to the tight junction found in vertebrate epithelia, the SJ provides a critical block to the paracellular flow of solutes and therefore allows tissue to form unique compartments for organ functions and organism protection. Two molecularly distinct versions of the SJ are present in *Drosophila* (and other invertebrate organisms) that differ in their ultrastructural appearance and molecular composition: smooth SJs (sSJs) and pleated SJ (pSJs). sSJs are present in the midgut and other endodermally-derived epithelia, and are comprised of the proteins Snakeskin, Mesh, Tetraspanin 2A, Fasciclin III (FasIII), Coracle (Cora), Discs Large (Dlg) and Lethal (2) Giant Larvae (Lgl). sSJs lack the electron-dense septa found in the pSJs of ectodermally-derived epithelia. An excellent review on the composition and function of the sSJ can be found at [2]. The remainder of this review will focus on the pSJ, and we will refer to it simply as the SJ.

Studies in *Drosophila* have revealed a few dozen genes that function in the establishment or maintenance of SJs and have provided insights into the biogenesis of the junction during embryogenesis and in the follicular epithelium of the ovary. Interestingly, mutations in many of the genes that encode core SJ proteins show defects in developmental and cellular processes that suggest that these proteins have functions that may be independent of their roles in providing an occluding junction. In this review, we will describe the known composition of SJs, the biogenesis of the SJ in embryonic ectodermal epithelia, and the functions that core SJ proteins likely play outside of their role in forming the occluding junction. We apologize to any colleagues for omissions of their work in this area as we endeavor to highlight non-occluding functions of SJ proteins and are not necessarily providing an exhaustive catalog of functions ascribed to SJ proteins.

## 2. Structure and Composition of SJs

**Figure 1:**
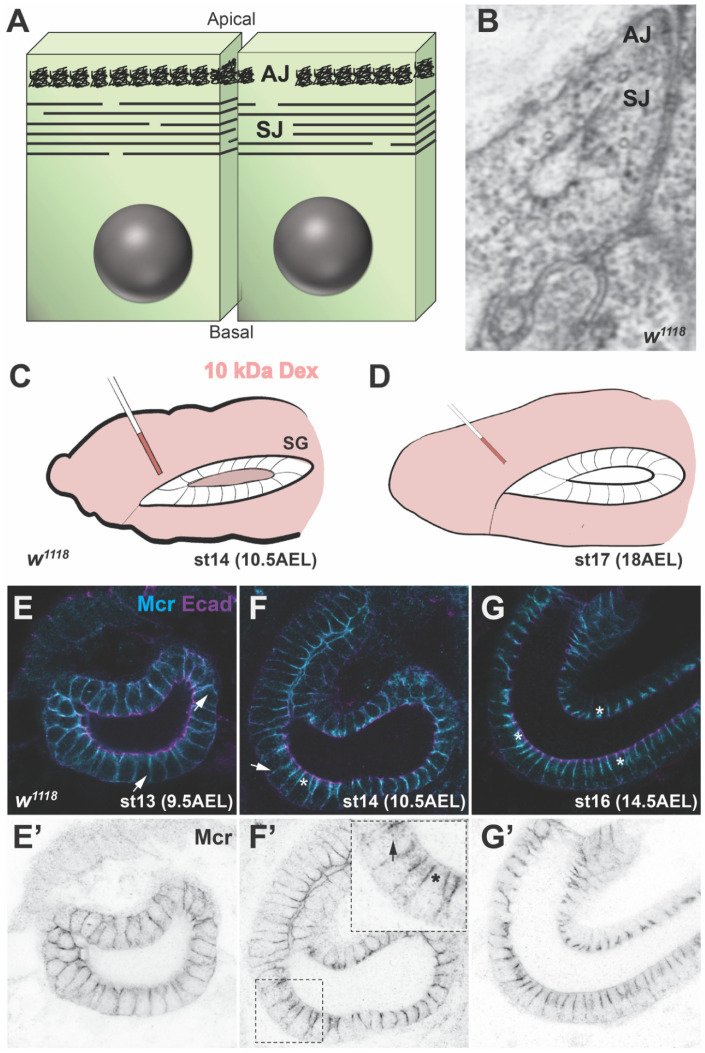
Structure and biogenesis of SJs. (**A**) Diagram of a polarized epithelium, showing the location of the adherens junction (AJ) and the septate junction (SJ). Apical is to the top. Note that the septa do not completely encircle the cell but are discontinuous, generating an extended path from the apical to basal side of the epithelium. (**B**) Electron micrograph of the epidermis in a stage 17 wild type embryo showing the electron dense extracellular septa in the SJ. (**C**,**D**) Diagram of the anterior portion (including a salivary gland) of a stage 14 (**C**) and a stage 17 (**D**) wild type embryo that had been injected with a 10-kDa rhodamine-labeled dextran (pink). The labeled dextran can cross the salivary gland epithelium and enter the lumen in a stage 14 embryo, but not in a stage 17 embryo due to the formation of the SJ. (**E**–**G**) Confocal micrographs of hindguts from *w^1118^* stage 13–16 embryos stained with antibodies against the AJ protein E-cadherin (in magenta) and the SJ protein Mcr in cyan. (**E’**–**G’**) Inverted images of the Mcr channel from (**E**–**G**). Mcr localizes all along the lateral membrane in stage 13 embryos (arrows) and begins to be enriched at the SJ (asterisk) with some lateral expression (arrow) and punctate cytoplasmic expression in stage 14 (arrow in inset). By stage 16, Mcr is tightly localized to the region of the SJ (asterisks) and is clearly basal to the AJ protein E cadherin.

SJs are found in ectodermally-derived epithelia in invertebrates, including the epidermis, the fore- and hind-gut, the salivary glands and the trachea of the embryo, as well as imaginal discs in larvae and pupae [3,4,5]. In the adult gonads, SJs are present in the follicular epithelium of late-stage egg chambers [6] and in somatic cells surrounding the germline at late spermatocyte stages in the testes [7]. Finally, SJs have been observed in the encapsulation epithelium surrounding parasitic wasp eggs in larvae [8]. SJs localize to the apical lateral region of epithelial cells, immediately basal to the adherens junction (Figure 1). SJs appear as uniformly spaced rows of electron dense septa between the plasma membranes of adjacent cells by transmission electron microscopy [4]. Freeze-fracture and lanthanum infiltration electron microscopic analyses reveal that the septa are discontinuous in the plane of the membrane and do not completely encircle the cell. Thus, the septa provide a “tortuous, maze-like pathway” between the apical and basal sides of an epithelium [9].

The SJ can be further differentiated between the bicellular SJ that connects two adjacent cells, and the tricellular SJ (tSJ) that forms at the vertex of three epithelial cells. The tSJ is composed of unique proteins that include Gliotactin (Gli) [10], Bark Beetle (also known as Anakonda) [11,12], and M6 [13]. Readers interested in the organization and biogenesis of the tSJ are directed to the review by Higashi and Chiba [14], and recent papers by Esmangart de Bournonville [15] and Wittek [16].

The SJ serves to prevent paracellular flow between the apical and basal sides of an epithelium, much as tight junctions (TJ) provide a barrier function in vertebrate epithelia [1,17]. This function is typically demonstrated by the ability of the SJ to prevent the infiltration of a 10 kD dextran into the lumen of embryonic salivary glands, trachea or hindgut ([18]; Figure 1C,D). This function is critical for organ function, including germline differentiation in the testes by providing an insulating barrier to the spermatocytes by the somatic epithelium [19], and in establishing the blood-brain barrier through SJ formation in glial cells [20].

Genetic studies in *Drosophila* have identified at least 33 genes that are required for the establishment or maintenance of SJs (Table 1). These genes encode proteins that fall into three categories: core functional constituents of the junction, other SJ resident proteins, and accessory proteins required for SJ formation. Core SJ proteins are required for the establishment and maintenance of the junction *AND* physically reside in the junction. Examples of core SJ proteins include the transmembrane proteins Neurexin IV (Nrx-IV) [20], Neuroglian (Nrg) [21], Macroglobulin complement-related (Mcr) [22,23], Sinuous (Sinu) [24], and Kune-Kune (Kune) [25], the GPI-linked protein Contactin (Cont) [26], and the cytoplasmic proteins Cora [27] and Varicose (Vari) [28] (Table 1). SJ resident proteins physically localize to mature SJs, but are not required for their assembly or maintenance. Examples of SJ resident proteins include Dlg [29], Scribble (Scrib) [30] and Yurt (Yrt) [31] (Table 1). SJ assembly proteins are required for the establishment or maintenance of the junction, but do not reside in the junction. Examples of SJ assembly proteins include Leukocyte antigen 6 (Ly6) proteins such as Boudin (Bou), Coiled (Cold), Crimpled (Crim) and Crooked (Crok) [32,33,34,35], the lipid phosphatases Wunen and Wunen-2 (Wun and Wun2) [36], and proteins involved in endocytosis and recycling, including Clathrin heavy chain, Rab5 and Rab11 [37].

Studies in *Drosophila* are beginning to reveal the interactions that hold the SJ complex together within and between cells. Nrx-IV, Cont, Nrg and Lac are transmembrane or GPI-linked membrane proteins that contain combinations of extracellular domains involved in protein-protein and protein-matrix interactions, including Immunoglobulin, Epidermal Growth Factor-like, Lamin G, Fibronectin III, C-type lectin-like and Coagulation factor 5/8 C-terminal domains. In vivo structure/function analysis indicates that a LamG-EGF-LamG motif in Nrx interacts with Cont [39], suggesting that these proteins may form a heterophilic interaction that holds adjacent cells together at the junction. Similarly, Mcr is a transmembrane protein with a complete extracellular α2-macroglobulin domain. α2-macroglobulins in invertebrates function as a homodimer raising the possibility that Mcr may also function in adhesion at the SJ [23]. Lac also is capable of homophilic adhesion, as demonstrated by an in vitro Lac coated bead aggregation assay [41]. Functional SJs also require specific Na/K-ATPase subunits (Nervana2 and ATPα), but do not require ATPase catalytic activity, suggesting a structural role for these proteins in the SJ [50]. Three core SJ proteins are in the claudin superfamily, but while related claudins in tight junctions are thought to be involved in homo and heterophilic interactions in the tight junction [51], the size of their extracellular loops and the spacing between cells at the SJ would likely preclude them from providing a trans adhesive role in the SJ. In vitro and co-immunoprecipitation experiments indicate that the N-terminal FERM domain of Cora interacts with the cytoplasmic domain of Nrx-IV [52]. Similarly, in vitro binding and in vivo co-immunoprecipitation studies indicate that the PDZ domain of Vari interacts with the cytoplasmic domain of Nrx-IV [44]. How all of these proteins interact, and whether the composition and stoichiometry is conserved in all tissues remains to be determined. A mass spectroscopy analysis of proteins that co-immunoprecipitate with Mega from embryonic lysates, however, has been invaluable for finding new SJ proteins [12,40,46] and will likely provide additional insights into the composition and connections between SJ proteins.

## 3. Biogenesis of the SJ

The formation of the SJ during embryogenesis occurs in a step-wise fashion over several hours (Figure 1E–G). Many of the core SJ proteins are expressed early in embryonic development, and essentially all of them are strongly expressed by stage 12 of embryogenesis [53]. The initial localization of these proteins is uniform along the entire lateral membrane but is excluded from the very apical membrane. Beginning at stage 13, SJ proteins can be observed in intracellular puncta that co-localize with Rab5 and Rab11 (signifying early and recycling endosomes, respectively) [37], while the bulk of the proteins remain along the lateral membrane. Recycling of SJ proteins results in their gradual enrichment in an apical lateral region (the presumptive SJ) during stages 14 and 15. By the end of stage 15, a SJ protein accumulation is obvious at the presumptive SJ, although some SJ protein is still apparent along the lateral membrane. Finally, at stage 16, SJ proteins become tightly localized to the region of the SJ and are noticeably absent from the lateral membrane. This gradual assembly of the SJ complex is consistent with ultrastructural studies that show a stage-specific maturation of the junction with only a few dispersed electron dense septa in stage 14 embryos, followed by increasing numbers and a regularly spaced pattern of septa by stage 17 [5]. Consistent with this late organization of the SJ, functional studies using a 10 kDa labeled dextran indicate that the paracellular barrier is not established until late stage 15 in wild type embryos [38].

The process of SJ biogenesis requires that all core SJ proteins are present and functional. It was shown early on that SJ proteins are interdependent for their localization to the junction as loss of function mutations in any core SJ protein prevents the SJ localization of other core SJ proteins [21,52]. Likewise, mutations in any core SJ protein result in epithelia that are permeable to a 10 kDa dextran (e.g., [18,21,22,42]). Consistent with a leaky junction, mutations in core SJ proteins lack the electron-dense septa seen by electron microscopy in late stage embryos [22,37,42], although in some cases there is residual electron dense material in the intermembranal space, suggesting that junction formation had begun, but failed to complete [21,24,39].

A number of accessory proteins are also required for the formation of a mature SJ. As described above, SJ proteins appear to be endocytosed and recycled to the membrane. That this step is essential is validated by the defective SJ biogenesis in *rab5^DN^* (dominant negative) and *rab11^DN^* mutant embryos, in *Clathrin heavy chain* mutant embryos and in the *shibire* temperature-sensitive mutant at the restrictive temperature [37,49]. Similarly, loss of function mutations in any of the Ly6 genes *bou*, *crok*, *cold* or *crim* result in defective SJ formation [32,33,34,35,36]. Ly6 proteins are small (~150 amino acid) GPI-linked proteins with a single three-fingered protein domain stabilized by disulfide bonds. The *Drosophila* genome encodes at least 18 Ly6-like proteins, four of which appear to be required for SJ organization [34]. *bou* encodes a secreted protein that acts non-cell-autonomously in SJ organization [33], whereas Cold and Crok appear to reside in internal membrane compartments and function cell autonomously, suggesting that they may function in the vesicle trafficking or assembly of macromolecular SJ complexes en route to the plasma membrane [32,34]. *wun* and *wun2* encode transmembrane proteins with lipid phosphate phosphatase activity [36]. Wun localizes to the apical surface and to SJs, whereas Wun2 localizes more broadly in the cell, including intracellular membranes (based upon a myc-tagged recombinant protein). Maternal/zygotic (M/Z) mutations in *wun*/*wun2* disrupt the organization and function of SJs, although septa are observed by electron microscopy. How phospholipid composition affects SJ organization and function is unknown, although the apical accumulation of the putative chitin deacetylases Serpentine (Serp) and Vermiform (Verm) in tracheal cells is disrupted in *wun/wun2* M/Z embryos, raising the possibility that it may involve polarized trafficking of SJ proteins.

Fluorescence recovery after photobleaching (FRAP) experiments reveal that SJs are remarkably stable in the plane of the membrane [49,54]. As described by Oshima and Fehon [49], FRAP of SJ proteins is rapid in the epidermis of wild type stage 12 embryos, indicating that an immobile complex has not yet formed. The mobility of SJ proteins is reduced starting in early-mid stage 13 and reaches the maximum degree of immobility by late stage 13. Interestingly, SJ protein mobility can be restricted along a membrane in contact with one cell but remain mobile along a membrane in contact with a different cell. Thus, it seems that the protein immobility is due to interactions across the membrane rather than in the plane of the membrane, and that those between-cell interactions begin in stage 13. Remarkably, the loss of any core SJ protein results in the failure to generate an immobile SJ complex in the membrane. Since not all SJ proteins are capable of direct intercellular interactions (e.g., the cytoplasmic protein Cora), this suggests that stable interactions *within* the plane of the membrane are requisite to form stable interactions between complexes on adjacent cells.

Biogenesis of the SJ in the follicular epithelium of ovarian egg chambers follows a similar progression to that in embryonic epithelia. Core SJ proteins are localized all along the lateral membrane in stage 2–10 egg chambers, but are relocalized to an apical lateral location during stages 11 and 12. This relocalization of SJ proteins requires the presence of other SJ proteins, as RNAi against *Nrx-IV* or *Mcr* result in mislocalization of SJ proteins in stage 12 egg chambers [55]. Likewise, expression of a dominant negative Rab5 in the follicular epithelium or expression of *rab11-RNAi* results in mislocalized SJ proteins in stage 11/12 egg chambers. This progression of SJ biogenesis is consistent with ultrastructural analysis showing electron dense septa in the follicular epithelium of egg chambers from stage 10B, but not before ([6]; but note nascent electron dense material can be observed as early as stage 6: [56]).

## 4. Developmental Functions of SJ Genes

As mentioned above, loss of the occluding junction in embryonic epithelia and glia results in embryonic lethality associated with paralysis due to a defective blood-brain barrier [20]. Yet many of the SJ genes were originally discovered via genetic screens for developmental mutations, suggesting that these proteins may have additional functions in development. For example, several core SJ genes were identified in screens for mutations in tracheal morphogenesis [22,24,28,38,41,42,57], and *Mcr* was discovered in a screen for genes involved in imaginal disc morphogenesis during metamorphosis [23]. Accumulating evidence indicates that SJ proteins are required for developmental events that occur throughout the life of the fly. Below, we describe the evidence linking SJ gene function to distinct developmental processes.

### 4.1. Dorsal Closure (DC) and Head Involution (HI)

HI and DC are dynamic tissue migrations that occur between stages 13 and 15 of embryogenesis. Immediately after germ band retraction in wild type embryos (stage 12), the epidermis covers the ventral and lateral regions of the embryo leaving a dorsal hole plugged by an extraembryonic epithelium known as the amnioserosa (AS). At this stage of development, the six segments that will give rise to the head (3 procephalon and 3 gnathal segments) are found at the anterior of the embryo. Coordinated tissue movements in the epidermis and AS result in the migration of the head segments dorsally and towards the posterior to internalize the head segments beneath the dorsal ridge that forms during DC [58]. At the same time, the dorsal-most row of epithelial cells is organized by a Jun *N*-terminal kinase (JNK) signaling cascade to elongate in the dorsal-ventral axis and to produce an actomyosin contractile ring within these cells to coordinate movement of the lateral epidermis towards the dorsal midline. Pulsed contractions of medioapical actin fibers in the AS and cell shape changes in the epidermis promote the migration of the lateral epidermal sheets dorsally [59,60,61], until the contralateral epithelial sheets meet and fuse at the dorsal midline. DC is complete and HI nearly so by the end of stage 15 of embryogenesis.

It was recognized with the characterization of the first few SJ genes that DC and HI defects comprise some of the phenotypes associated with strong loss of function mutations. For example, *cora* was originally named for its dorsal open mutant phenotype (coracle being a small round boat) [27], and the authors also noted HI defects in *cora* mutant embryos. Subsequently, Ward and colleagues [62] showed that approximately 40% of *cor^5^* mutant embryos (a null allele) failed to complete DC and that this phenotype could be rescued by expression of either a full-length *cora* transgene or just the N-terminal FERM domain. Similarly, Baumgartner and colleagues [20] reported DC defects in *Nrx-IV* strong loss of function mutations, and Schulte and colleagues [10] noted that DC is delayed in some *Gli* mutant embryos (but appears to recover later in development as dorsal holes are not observed in the cuticles of dead *Gli* mutant embryos). The initial characterization of *Mcr* also revealed DC defects in mutant embryos [23].

**Figure 2:**
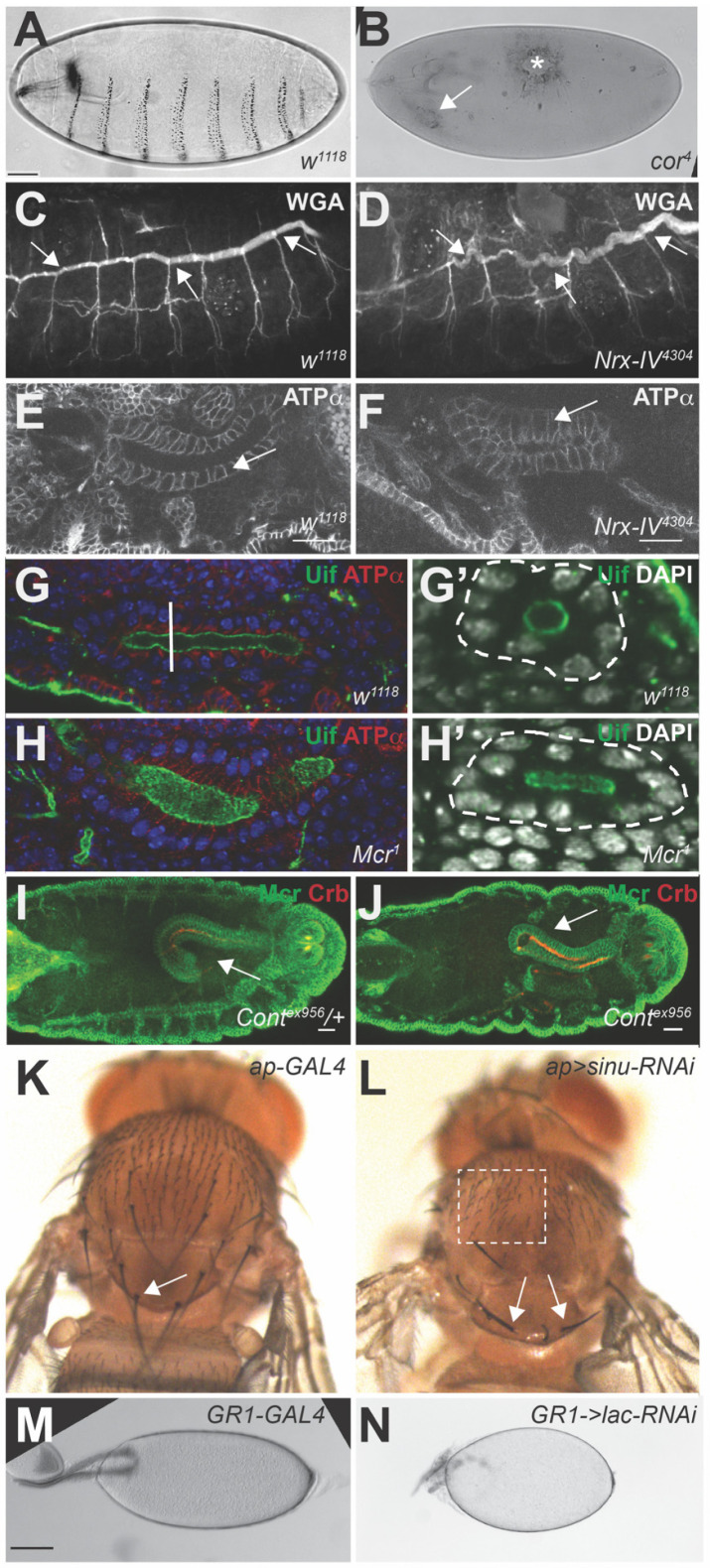
Embryonic phenotypes associated with SJ mutations. (**A**,**B**) Cuticle preparations of *w^1118^* (**A**) and *cor^4^* (**B**) embryos showing defective dorsal closure (asterisk in (**B**)) and extra cuticle deposits in the region of the salivary gland (arrow in (**B**)). (**C**,**D**) Wheat germ agglutinin stained stage 17 wild type and *Mcr^1^* embryos showing the long and convoluted *Mcr^1^* trachea (arrows). (**E**,**F**) Confocal images of salivary glands from *w^1118^* and *Nrx-IV^4304^* stage 16 embryos stained for ATPα to outline cells. The *Nrx-IV* gland is short and fat, with longer apical-basal dimensions (arrows). (**G**,**H**) Confocal sections of Z-series through salivary glands from stage 16 *w^1118^* (**G**) and *Mcr^1^* (**H**) embryos stained with antibodies against Uninflatable (green) and ATPα (red). DAPI is in blue. White line indicates cross section shown in (**G’**) and (**H’**) showing the morphology of the lumen. In *w^1118^* SGs there are 7.5 ± 0.3 nuclei around the lumen compared to 9.2 ± 1.1 in *Mcr^1^*. (**I**,**J**) Confocal images of hindguts from *Cont^ex956^/+* and *Cont^ex956^* stage 16 embryos stained for Mcr and Crb. The *Cont* hindgut has much less curvature than the wild type gut (arrows). (**K**,**L**) Dorsal thoraces of a control adult (**K**) and adults expressing RNAi against *sinu* (**L**) in the dorsal wing disc using *ap-GAL4*. Note the misoriented bristles and overall smaller scutellum and the *sinu-RNAi* thorax (arrows), and the overall disorganization of the bristles in the dorsal thorax (boxed in region). (**M**,**N**) Brightfield photomicrographs of *GR1-GAL4* (control) and *GR1>lac-RNAi* stage 14 egg chambers. Note that the lacRNAi egg chamber is substantially rounder than the control egg chamber.

The observation that mutations in several SJ genes affect DC and tracheal morphogenesis (see below), motivated Hall and Ward to undertake a reevaluation of nine core SJ genes for defects in DC, HI and organogenesis [53]. Mutations in all nine SJ genes showed highly penetrant defects in HI (ranging from approximately 50% to 100%), while many showed some degree of DC defects that could be observed by cuticle preparations (e.g., Figure 2B). The mutations that showed the most penetrant defects in DC were in *Nrx-IV*, *cora* and *Mcr*.

It is informative to correlate the timing of DC and HI with the steps involved in SJ biogenesis (Figure 1E–G). HI and DC both initiate at the end of germ band extension, a time at which all of the SJ proteins are expressed, but are evenly localized all along the lateral membrane. As HI and DC progress in stage 13 and 14, SJ proteins are beginning to be internalized and recycled back to the membrane (Figure 1F). As HI and DC are nearing completion in late stage 14/early stage 15, SJ proteins are enriched at the prospective SJ (with some of the protein still localized along the lateral membrane) and have become immobile in the membrane. DC is complete and HI nearly so before the junction is physiologically tight (impermeable to a 10 kDa dextran), and both processes are finished before the SJ looks mature by ultrastructure. Together, these observations suggest that SJ proteins have a role in morphogenesis that is independent of their role in forming an occluding junction. That common developmental defects are found in essentially every core SJ mutant argues against a pleiotropic effect for one or a small number of these proteins, and instead suggests that these proteins function together in these developmental contexts.

### 4.2. Organogenesis of the Trachea, Salivary Glands and Hindgut

The majority of the core SJ genes were discovered due to a long and convoluted embryonic trachea defect (e.g., Figure 2D), making this the most diagnostic developmental phenotype associated with SJ genes. Tracheal morphogenesis begins with placode formation of each of the bilateral tracheal metameres (unit elements of the tracheal system that form in thoracic segment 2 through abdominal segment 8) during stage 9. Each placode begins invaginating during germ band extension (stage 11), undergoes stereotypic branching during stages 12–13, and fuses to form a continuous trachea with a common lumen in the dorsal trunk during stage 15 (reviewed in [63]). Control of tracheal length in the dorsal trunk is mediated via at least five independent pathways that appear to function between stages 14 and 16. These pathways include the lumenal accumulation of Serp and Verm [64,65], maintenance of apical-basal polarity [31], planar polarized anisometric growth mediated through *Src42* [66], cell adhesion or matrix organization [57], and a non-canonical Hippo pathway involving Yorkie activation of Diap1 [67]. Defects in any of these mechanisms lead to dysregulation of the apical relative to basal surface in tracheal dorsal trunk cells resulting in either large and convoluted or short trachea. Two of these mechanisms appear to require the function of SJ proteins. First, zygotic loss of several core SJ gene results in a failure to accumulate Serp and Verm in the tracheal lumen (e.g., [22,25,28,46]), leading to convoluted trachea. Second, *cora* acts redundantly with *yrt* to oppose the apical determinant *crumbs* (*crb*) and maintain apical-basal polarity in the trachea [31]. This function of *cora* in trachea length control is similar to its role (along with several other SJ genes) in maintaining apical-basal polarity in the epidermis (more on this below) during mid-embryogenesis [68]. Interestingly, tracheal length defects in *cora*, *yrt* double mutants can be rescued by loss of *crb*, but this does not rescue the Serp and Verm accumulation defect, indicating that these mechanisms are independent [31]. Although the timing of tracheal length control overlaps with the timing of occluding junction formation (stage 15–16), there are a couple of reasons to suggest that the function of SJ proteins in these processes are independent of their occluding function. First, there is evidence of SJ mutations that clearly fail to form an occluding junction, but still maintain normal tracheal length [38]. In addition, there appear to be at least some SJ mutations that result in convoluted trachea, but do not alter apical-basal polarity in mid-embryogenesis [25].

Morphogenesis of the salivary gland begins in a similar manner to that of the trachea with the formation of bilateral placodes on the ventral surface of parasegment 4 during stage 10 (reviewed in [63]). During stages 12–15, the salivary gland primordia migrate inwardly until the tips reach the visceral mesoderm, at which point they turn and migrate posteriorly. During their migration, salivary glands elongate (along the anterior/posterior axis) through a process involving cell shape changes and cell rearrangements [69,70,71]. The first observation suggesting that SJ genes are required for some aspect of salivary gland development was the discovery of abnormal deposits in the region of the salivary glands in cuticle preparations of *cora* and *Nrx-IV* mutant embryos ([52]; e.g., Figure 2B). These phenotypes were also observed in *Nrv2*, *Atpα*, *Nrg*, and *Gli* mutations by Genova and colleagues [21], who additionally noted that the morphology of these glands was abnormal. Similar defects observed in the salivary glands of *Mcr* mutant embryos [23] led Hall and Ward to examine salivary gland morphogenesis in *Cont*, *kune*, *Mcr* and *Nrg* mutant embryos in greater detail [53]. In all of these mutants, the glands were observed to be short and fat with irregular lumens often seen as flattened (e.g., Figure 2F). In addition, many of the glands showed a bent morphology suggesting a defect in the migration of the gland along the visceral mesoderm. By counting the number of nuclei around the lumen of wild type and SJ mutant glands between stages 11 and 16 and by measuring the length of the lateral membranes in these SGs, they showed that these defects were due to a reduction in the cell rearrangements that lengthen the gland during development, and to a lesser degree by an apical-basal cell shape change that normally converts the tall columnar salivary gland cells into a more cuboidal form (e.g., Figure 2H). That these defects can be observed in embryos as early as stage 13 again suggests that this potential function for SJ proteins is independent of their function in the formation of the occluding junction.

A potential role for SJ proteins in hindgut morphogenesis was uncovered by the observation of straight (rather than curved) hindguts in *FasIII* mutant embryos [72]. In wild type embryos, asymmetric activation of JAK/STAT signaling in the cells that constitute the inside curve of the hindgut lead to enhanced expression of FasIII in these cells from stage 12 to 15. This highly expressed FasIII protein localizes all along the lateral membrane in cells inside the curve, even as FasIII localizes to the presumptive SJ in the cells that make up the remainder of the hindgut in stage 14/15. Given FasIII can serve as a homophilic adhesion molecule in S2 cells and in wing imaginal disc clones [72,73], the authors proposed that FasIII-mediated enhanced cell adhesion in the inside curve is responsible for hindgut curvature. Consistent with this notion, ectopic overexpression of FasIII throughout the hindgut leads to lateral FasIII in all hindgut cells, and straighter hindguts. Although FasIII is considered a SJ-associated protein and not a core component of the junction, the author went on to show that mutations in the core SJ gene *Varicose* also showed straightened hindguts (e.g., Figure 2J). Again, the timing of these morphogenetic events, and the fact that the mechanism of hindgut morphogenesis appears to involve asymmetric cell adhesion suggest a function for SJ proteins that is independent of the occluding junction.

### 4.3. Dorsal Vessel Development

Somewhat surprisingly, many SJ proteins are also required for the development of the dorsal vessel (heart), even though there is no SJ formed in this tissue [74]. The *Drosophila* embryonic heart is a linear organ composed of two rows of cardiomyocytes, the cells which contract to pump hemolymph through the organism, surrounded by two layers of pericardial cells, which have secretory, regulatory, and filtration roles [75,76,77,78,79]. Although SJs are not found in the dorsal vessel, two clear roles within the dorsal vessel have emerged for SJ proteins: in cell-cell adhesion and as effectors of reactive oxygen species-mediated paracrine regulation of cardiac function [77,80].

The role of SJ proteins in adhesion was initially identified as a result of a screen for genes resulting in heart defects [81]. This screen identified several mutants that result in separation of the cardiomyocytes from the pericardial cells, a phenotype termed “broken hearted.” Analysis of these mutants revealed that the affected genes included those encoding HMG-CoA reductase and G protein γ subunit Gγ1, as well as *Nrx-IV* [80,81]. Additional study revealed that *Cont*, *cora*, *Gli*, *Lac*, *Nrg*, *nrv2*, and *sinu* are also required for adhesion and result in the broken hearted phenotype when mutated, with a lower penetrance for *Gli*, *Lac*, and *Nrg* [80]. Double mutant analysis of *cora*, *Nrx-IV*, *nrv2*, and *sinu* with *Gγ1* demonstrated that these SJ proteins act in a pathway with Gγ1. In *Gγ1* mutants, Nrx-IV expression is dramatically reduced and Cora, Sinu, and possibly Nrv2 are mislocalized. As in situ hybridization suggests that *Nrx-IV* mRNA is not altered in *Gγ1* mutants, these results suggest that Gγ1 is involved in post-transcriptional regulation of *Nrx-IV*, which in turn is required for correct localization of other SJ proteins and proper cell adhesion in the dorsal vessel. Mutation of any of the genes encoding these proteins also results in reduced heart rate during late embryonic stages suggesting that cardiac performance is also inhibited.

In addition to the adhesive roles played by this wide set of SJ proteins, a critical role in regulating the structure and function of the dorsal vessel has been identified for a much smaller set of SJ proteins: Cora and the claudin Kune. These proteins were identified as sensitive to levels of reactive oxygen species (ROS) in pericardial cells, a characteristic not possessed by any of the other SJ proteins tested, nor by other members of the Protein 4.1 family [77]. The ROS signaling cascade acts through proteins such as the p38a MAP kinase in the pericardial cells to regulate critical processes in the neighboring cardiomyocytes [82]. Cora acts downstream of p38a and relies upon p38a for proper levels of protein expression [77]. In turn, Cora regulates expression of Kune, with higher levels of Cora resulting in lower levels of Kune and vice versa. Unlike the localization of SJ components in epithelial tissues, Cora and Kune are not co-dependent for expression in pericardial cells: increasing or decreasing Kune levels has no effect on expression of Cora. However, as a claudin, Kune is a transmembrane protein and appears to regulate Kune levels in neighboring cells in the dorsal vessel: increasing or decreasing Kune specifically in cardiomyocytes leads to a similar increase or decrease in Kune in pericardial cells and vice versa. This paracrine regulation of Kune levels in the heart does not occur in epithelial tissues, consistent with the notion of a distinct, non-canonical role for Cora and Kune in the dorsal vessel. The exact level of these proteins appears to be critical for normal cardiac function: overexpressing or underexpressing Kune or its upstream regulators results in altered heart period, arrhythmicity, and decreased diameter, phenotypes which are relieved by 3 weeks of age if Kune is overexpressed but persists if Kune is underexpressed. These phenotypes in turn may result from altered filtration of hemolymph by pericardial cells: increased or decreased levels of Cora or Kune often result in smooth cells that lack labyrinthine channels and slit diaphragms that are believed to be associated with the filtration function of these cells [83].

### 4.4. Imaginal Disc Morphogenesis

SJ proteins have also been implicated in a number of morphogenetic processes that occur in imaginal discs during metamorphosis. Imaginal discs form as involutions of the embryonic epidermis. They grow and are patterned during larval stages, and then undergo complex morphogenetic movements to give rise to the adult integument during metamorphosis [84]. The imaginal epithelium contains SJs that are established during embryogenesis and appear to be remarkably stable, even within cells undergoing mitosis [49,54,84]. Since strong loss of function mutations in all core SJ genes result in embryonic lethality, the interrogation of SJ protein function during metamorphosis has relied mostly on hypomorphic alleles and targeted expression of RNAi constructs against these genes and thus likely underestimates their function in these developmental events. A genetic screen for *cora* alleles identified three hypomorphic alleles that are either fully adult viable (*cora^15^*) or produce a small percentage of adult escapers (*cora^7^* and *cora^14^*; [18]). Phenotypes associated with these homozygous *cora* mutant adults include malformed legs and rotation defects in the genitalia and ommatidia [18]. Hypomorphic mutations in *Gli* produce similar leg defects [10]. Adults heterozygous for *Mcr^1^* in a *broad^1^* mutant background also show malformed legs with short, fat tarsal segment [85]. These leg phenotypes are suggestive of a defect in the cell shape changes and rearrangements that occur during the prepupal stage [86,87]. Hypomorphic *Gli* and *cora* mutant adults also display misoriented wing and thorax bristles (that are thinner and frequently bent) (e.g., Figure 2L), likely due to a process that normally occurs in late pupal wings [88]. Consistent with these observations, genetic reduction of *vari* in imaginal discs via RNA interference or by an incomplete rescue of a null *vari* mutation (using the ubiquitous daughterless GAL4 to drive *vari* cDNA) produced misoriented wing hairs in the adult, along with rough eyes due to missing ommatidia or extra interommatidial bristles [45]. These authors also mentioned that the adults were unable to walk, although a description of any leg malformations was not mentioned.

### 4.5. Morphogenetic Events during Oogenesis

A recent study has revealed roles for several SJ proteins in morphogenetic events that occur during oogenesis [55]. The ovaries in adult females are composed of approximately 20 ovarioles, each of which consists of linked chains of successively more developed egg chambers. An egg chamber consists of a 16-cell germ-line-derived cyst (1 oocyte and 15 nurse cells) surrounded by a somatic follicular epithelium. Egg chamber maturation occurs over 14 developmental stages (reviewed in [89]). The follicular epithelium has apical-basal polarity with junctional organization on the lateral membranes. Mahowald [6] demonstrated the presence of mature SJs beginning in stage 10B egg chambers using electron microscopy, although there is evidence of some nascent SJ extracellular material as early as stage 6 [56]. Not surprisingly, many of the core SJ proteins have been identified in the follicular epithelium, consistent with the presence of SJs in this tissue [23,90,91,92,93]. A number of important morphogenetic events occur during egg chamber development, including egg elongation, dorsal appendage formation, and border cell migration [89,94]. Using pan-follicle cell RNAi against *Mcr*, *Nrx-IV*, *Cont*, *cora*, *sinu*, and *lac*, Alhadyian and colleagues [55] demonstrated that these core SJ genes are required for the establishment or maintenance of an elongated egg shape (e.g., Figure 2N). They went on to show defects in the morphology of the dorsal appendages in all but the *sinu-RNAi* egg chambers. Given the timing of these events, it is unclear if the defects seen in these SJ-RNAi-induced egg chambers are due to a loss of the occluding junction, or if they result from an independent function of these proteins. RNAi against *cora*, *Nrx-IV* and *Cont* in border cells led to mild defects in border cell migration characterized by incomplete migration, with some border cell clusters failing to delaminate at the beginning of the process or disassociating during the migration [55]. In this case, the defective phenotypes occur prior to the formation of a mature occluding junction, arguing for an independent (although unknown) function for SJ proteins in this process.

### 4.6. Hemocyte Encapsulation of Parasitic Wasp Eggs

Interestingly, SJ proteins are expressed in migrating hemocytes, and are essential for an effective response to parasitic wasp attack. In the larva, embryonic-derived hemocytes expand in population size and differentiate into three cell types: plasmatocytes, lamellocytes and crystal cells [95]. These cells are sessile and generally act as individual cells. In response to being parasitized by a wasp, however, lamellocytes expand in number and converge on the egg, eventually forming an epithelium around the egg. This encapsulation epithelium forms bona fide SJs within the epithelium to protect the larval tissue from collateral damage once the crystal cells degranulate to degrade the foreign egg [8]. A number of SJ proteins are known to be expressed in the sessile lamellocytes prior to encapsulation [96,97], although it is unknown if all SJ proteins are ubiquitously expressed or if adhesion or another signal initiates expression of the full complement of SJ proteins. Reduction of Nrg via RNAi results in failure to mount an encapsulation response [96], whereas *Rac* mutations result in reduced and mislocalized Cora expression that also prevents encapsulation [97]. It is unclear if there are functions for SJ proteins that are independent of forming an occluding junction in this system, but investigating the steps involved in the formation of an encapsulation epithelium may serve as an excellent model to study SJ biogenesis.

## 5. Cellular Functions of SJ Genes

Given the studies described above, it is clear that SJ proteins participate in a range of developmental events, and that their function in many of these events is likely independent of their role in creating an occluding junction. A few of these studies suggest potential cellular processes that require SJ proteins. For example, defects in embryonic salivary gland morphogenesis indicate a role for SJ proteins in cell shape changes and rearrangements [53], as do defects associated with loss of SJ proteins during metamorphosis [10,18,85]. Similarly, defects in hindgut morphogenesis reveal a potential role for SJ proteins in differential adhesion along the lateral membrane [72]. Below we review additional studies that investigate cellular processes that require SJ genes as a means to help illuminate potential roles that SJ proteins may play during development.

### 5.1. Biomechanical Tissue Regulation during Wound Healing

Wound healing has become an excellent model system to study cellular events in morphogenesis. Following a laser-induced wound in the epidermis of a late-stage embryo, polarized accumulation of actin and myosin along the edge of the wound results in the formation of a cable encircling the wound, similar to that found along the epidermal leading edge during DC [61,98]. This cable was initially proposed to act as a purse-string, drawing the wound closed as it contracted [61,98,99], but more recent studies have suggested that it does not provide significant contractile force and may instead maintain epithelial organization by distributing forces evenly along the wound margin [100]. Wound margin cells also extend actin-rich filopodia and lamellipodia involved in cell migration and sealing of the wound [98,99], while the cells more distant from the wound undergo shape changes and rearrangements to allow wound closure [101].

Many of the cellular events that occur during epithelial wound closure require SJ genes. A 2018 study by Carvalho and colleagues found that mutants for *sinu*, *kune, Nrx-IV, Nrg, cora, vari, cold, crok, Gli,* or *Dlg* all demonstrate frequent failure to close wounds induced at stages 15–16 of embryogenesis [102]. Close examination of *kune* and *sinu* mutants revealed that although an actin-myosin cable initially formed around the wound margin, the intensity of fluorescently labeled actin and myosin was reduced compared to wild type embryos. Some mutants exhibited a “mild” phenotype, in which the wound contracts continuously to close at a slower rate than wild type, whereas other mutant embryos showed a “strong” phenotype with fading of the actin-myosin cable and wound expansion. These defects may partially reflect interference with actin-myosin cable formation by mislocalized junctional components; Nrx-IV fails to be properly depleted from the wound margin and inappropriately colocalizes with actin in spots along the cable. Given the highly codependent localization of SJ components described above, such interference by mislocalized components could explain the breadth of SJ genes that give rise to wound closure defects. In addition, E-cadherin also fails to decrease at the wound margin in SJ mutant embryos, a process that occurs in wild type animals. E-cadherin clearance and overall effective wound closure requires endocytosis [103], raising the question of whether SJ proteins play a role in facilitating this endocytosis.

*kune* wound repair phenotypes also appear to result from tissue-level mechanical alterations [102]. When *kune-RNAi* is expressed in *engrailed* (posterior) stripes in the embryo, defective wound closure occurs even when wounds are made in areas that do not express RNAi against *kune*. Similarly, wounds that straddle the boundary between *kune-RNAi*-positive and -negative cells exhibit similar actin-myosin cable intensities in both regions. *kune* also appears to be required for cell rearrangements that normally occur near the wound margin. Together, these results suggest a non-cell-autonomous role for *kune* in regulating biophysical properties of the epithelium. Consistent with this, laser ablation of *kune* mutant epidermis results in faster relaxation times than in wild type epidermis, indicating either increased tension and stiffness or a decreased viscous drag coefficient. It is unclear whether these mechanical properties require a claudin-specific function or reflect a requirement for the entire SJ complex, although the large number of SJ components required for wound closure [102] suggests the latter.

### 5.2. Planar Cell Polarity in Imaginal Epithelia

Most epithelial tissues exhibit cellular organization and molecular asymmetries within the plane of the epithelium that is orthogonal to the apical-basal axis. This type of polarity is known as planar cell polarity (PCP). In *Drosophila*, classic examples of PCP include the precise arrangement of wing hairs towards the distal wing margin, and ommatidial orientation in the compound eye. In these examples, PCP is mediated by highly conserved components of the non-canonical Wnt/Frizzled (Fz) pathway, also known as the core PCP module. Each core PCP factor localizes in a planar polarized fashion at the adherens junction along the lateral membrane, establishing a spatial cue across adjacent cells and mediating local cell interactions [104]. Loss of PCP genes in these tissues (the larval imaginal discs) causes aberrant hair orientation and ommatidial rotation defects that can be observed in the adult. An additional polarity system consisting of the atypical cadherins Fat and Dachsous (Ft/Ds system) works with or in parallel to the core PCP pathway [105]. Other Fz-independent mechanisms regulating planar polarity have been identified in various developmental contexts. For example, axis elongation in the embryonic epidermis requires planar polarized non-muscle myosin II and Par-3 along the anterior-posterior and dorsoventral axes respectively, to coordinate cell shape changes, cell rearrangements and oriented cell divisions [106].

Several studies have indicated a role for SJ proteins in planar polarity events in imaginal tissues, some of which appear to be independent of the core PCP pathway. As mentioned above, hypomorphic *cora* mutations result in adults with rotation defects in the ommatidia and male genitalia [18], as well as misoriented wing and thorax bristles [88]. Similarly, loss of *vari* in imaginal tissues results in misoriented wing hair and ommatidial disruptions [45]. The authors suggested that the misalignment phenotypes in *vari* are likely due to altered cell polarization and not due to disruption of core PCP [45].

A thorough analysis of the role of *gli* and *cora* in the pupal wing epithelium revealed a requirement in planar polarization that is independent on the core PCP pathway [88]. Early in pupal wing development, Gli and Cora are restricted to tricellular junctions but are transiently remodeled after pre-hair initiation. Gli and Cora are gradually diffused from junctions and are redistributed in a planar polarized manner forming apical ribbons with Gli enriched along the proximal to distal vertices and Cora enriched along the anterior-posterior axis. Later in pupal development, Cora and Gli are lost apically but localize to the basolateral membrane. Partial loss of *gli* or *cora* in the pupal wing epithelium results in misalignment of hairs. The authors conclude that the role of Gli and Cora is independent of the core PCP pathway for several reasons including the fact that overall proximal distal hair alignment is normal in the SJ mutant tissue, while alignment of neighboring hairs is disrupted, which is the opposite of the phenotypes found in *fz* mutant tissue [88]. In addition, the wing prehairs initiate at the distal vertex in *Gli* mutant pupal wing cells, whereas *fz* and other core PCP mutations result in prehair formation from the cell center.

### 5.3. Apical-Basal Polarity in the Embryonic Epidermis

Apical-basal polarity is fundamental to the organization and function of epithelial tissues. Polarization events divide the plasma membrane into distinct apical and basolateral domains that are established during cellularization by complex interactions between different multiprotein complexes (reviewed in [107]). Genetic and cell biological studies in *Caenorhabditis elegans* and *Drosophila melanogaster* have identified sets of evolutionarily conserved polarity proteins that regulate apical and basal identity in epithelial cells. The apical domain is regulated by the *Partitioning defective* (*par*) group of proteins and the *crumbs (crb)* complex, whereas the *scribble* (*scrib*) group acts on the basolateral region. Notably, the *scrib* group proteins are SJ resident proteins (Table 1). Mutual antagonism mediated mainly by cross-phosphorylation of substrate proteins by kinases from the opposing polarity group helps to maintain segregation between the domains. For example, atypical protein kinase C (aPKC) localizes apically and excludes basolateral proteins via its phosphorylation activity, whereas basolateral Par1 kinase restricts apical markers.

Yrt, a band 4.1 Ezrin-Radixin-Moesin (FERM) protein plays a conserved multifunctional role in regulating epithelial polarity and restricting apical membrane growth. In early embryogenesis (stage 11), Yrt is localized to the basolateral membrane and is excluded from the apical domain by aPKC mediated phosphorylation. Beginning at stage 14, Yrt is gradually enriched apically where it physically interacts with Crb and inhibits its activity as a part of the Crb complex, preventing apical membrane overgrowth [108,109]. Interestingly, during stages when organogenesis is occurring (stage 12–15), *yrt* also promotes basolateral membrane stability by genetically interacting with three core SJ genes (*Nrx-IV*, *cora* and *ATPα*) to counteract apical domain expansion [68]. Single SJ mutants do not exhibit any major defects in epithelial polarity. However, stage 12–15 double mutants of *yrt* and *Nrx-IV* or *ATPα* display significant disruption in polarity, including mis-localization of Crb throughout the length of the basolateral plasma membrane and defects in tissue organization. Similarly, *yrt cora* mutants exhibit apical membrane defects that phenocopy *crb* overexpression. This suggests that *yrt, Nrx-IV, ATPα* and *cora* cooperate (known as the *yrt/cora* group) to maintain basolateral membrane stability by inhibiting *crb* activity during organogenesis stages. Although Yrt colocalizes with SJ components including Cora, Nrx-IV and ATPα, and is required for SJ barrier function, it is not required for SJ assembly [68]. Notably, during terminal differentiation (stage 17), an unknown basolateral polarity module has been proposed that functions independently of *scrib* and the *yrt/cora* group [68].

### 5.4. Lateral Membrane Adhesion

As described above, differential lateral membrane adhesion appears to drive hindgut curvature during embryogenesis [72]. But lateral membrane adhesion also plays a role in tissue homeostasis, as it is critical in several developmental contexts to maintain a monolayered epithelium should cells divide out of the plane of the epithelium. In the ovarian follicular epithelium, the early embryonic ectoderm and the pseudostratified neuroepithelium of the optic lobe, misaligned cell divisions can result in daughter cells that are born in positions that will fall out of the plane of the epithelium. Rather than undergoing cell death, these cells are reintegrated into the epithelium. This is an active process that requires the core SJ protein Nrg and FasII [110]. Both proteins are immunoglobulin-containing adhesion molecules, and act synergistically through a mechanism that seeks to maximize cell-cell adhesion along the lateral membrane. Full activity also requires the cytoplasmic domain of Nrg that interacts with the cytoskeletal-membrane linker protein Ankyrin [111]. Notably, this process does not happen in the imaginal disc epithelium, where instead misoriented divisions lead to basal extrusion and cell death [112]. One key difference between these developmental contexts is that in the early embryonic ectoderm and early-stage follicular epithelium (cell divisions cease in stage 6 egg chambers), SJs have not formed and Nrg and FasII are strongly expressed all along the lateral membrane, whereas the SJ has formed and these proteins are tightly linked to the apical lateral junction in imaginal tissues. Interestingly, reintegration (at least in the follicular epithelium) does not appear to require the full complement of SJ proteins, as knockdown of Nrx-IV does not lead to reintegration failure [111].

### 5.5. Polarized Secretion?

An intriguing phenotype found in many SJ mutants is defective organization of cuticular structures. These defects were initially described in *cora* mutant embryos where it was noticed that the ventral denticle belts were poorly differentiated, and that the epidermal cuticle delaminated between the procuticle and epicuticle layers [18]. These epidermal cuticle phenotypes are found in mutations for many of the core SJ genes [53]. Similarly, irregularities in the tracheal cuticle (taenidia) was observed by Wu and colleagues in *sinu* mutant embryos [24]. The authors also noted large numbers of the vesicles accumulating in the subapical region of the tracheal epithelium by electron microscopy. It has been well-documented that SJ proteins are required for the luminal accumulation of Serp and Verm in the trachea [22,25,28,46], although whether these proteins are not made, are not secreted, are mis-directed, or leak through the epithelium via the paracellular route has not been fully determined and needs further study. Wang and colleagues showed that at least some Verm protein is retained in the tracheal cells of *ATPα*, *lac* and *sinu* mutant embryos [65]. Interestingly, these potential secretion defects appear to be target specific as Gasp (the 2A12 antigen) is correctly secreted and retained in the tracheal lumen of these same mutant embryos [65]. A potentially related defect is the abnormal deposits observed in the region of the salivary glands (and occasionally trachea) in cuticle preparations from SJ mutant embryos including *Nrx-IV*, *cora*, and *Mcr* (e.g., Figure 2B) [18,23,52].

## 6. Concluding Remarks

In this review we have presented work describing the composition and biogenesis of epithelial SJs, and have shown that core components of the junction are required for developmental events throughout the life of the fly that suggest that they may have occluding junction-independent functions. Many questions remain to be answered about both the occluding and non-occluding functions of these proteins, but recent work suggests that we are in a great position to address them.

Although we now know more than 30 proteins that function in the establishment or maintenance of the SJ, we still likely do not have a full accounting of the composition of the junction, nor understand how the junction matures during its biogenesis. More open-ended screens specifically aimed at identifying SJ components will help close this gap. The co-immunoprecipitation/mass spectroscopy analysis of Jaspers [40] and the RNAi screen of Deligiannaki [43] are excellent efforts in identifying new core and accessory SJ genes and could be expanded by using additional tissue samples or different RNAi assays. These studies will allow us to address with more certainty which proteins are involved in intercellular interactions. Is it Nrx-IV and Cont, or Mcr, or a combination of several homo- and heterophilic interactions? Is there an unknown secreted protein that acts as an intermediary to glue two half SJs together (and is the basis for the electron dense septa)? How do SJ proteins interact within the cell? We know of interactions between Cora and Nrx-IV and between Vari and Nrx-IV, but what holds all of the proteins together to make the complex so immobile? And what are the steps in the assembly process? Do different SJ proteins form subcomplexes that then come together to form the higher ordered structure? What determines the timing of biogenesis? We are struck by the necessity of endocytosis and recycling for the maturation of the SJ. What is happening in the endosomes that makes SJ proteins competent to form complexes across the membrane? Is there sorting in the endosomes that allow them to form complexes in cis that promote competence for intercellular contact? Alternatively, could there be a biochemical event that promotes maturation? For example, could a drop in the pH of the endosome change the conformation of the extracellular domain of a SJ protein(s) that allows it to participate in intercellular interactions? What are the cellular functions of the accessory proteins? Does the phospholipid composition of the plasma membrane change during biogenesis, and what role does Wun/Wun2 or any other lipid modifying enzyme play in this process?

We similarly have work to do to better understand the non-occluding functions of SJ proteins, but are moving towards more clarity. By examining the function of core SJ proteins in a variety of different developmental contexts, we are starting to see some common themes. For example, it appears that SJ proteins are required for cell shape changes and rearrangements during DC, organogenesis, in response to wounding and during imaginal disc morphogenesis. Biophysical studies indicate that SJ proteins may function non-cell-autonomously to promote tissue-level properties, perhaps in membrane stiffness or regulating tension at junctions. Similarly, in a couple of different contexts, SJ proteins appear necessary for apical-basal polarity. Does this function contribute to a potential role in apical secretion? Does it affect the ability of cells to flatten during key developmental events (salivary gland morphogenesis, epidermal cell flattening at the end of DC, follicle cell flattening after nurse cell dumping during oogenesis?). More broadly, we are also interested to know how much of the SJ protein complex is required for these non-occluding functions. In some contexts, it appears that only a subset of SJ proteins is required (i.e., apical-basal polarity in the epidermis and trachea, and some aspects of dorsal tube formation). Do some of these proteins function redundantly, or in a tissue- or stage-specific manner? Finally, are these non-occluding functions of SJ proteins ancestral, and have these functions been maintained through evolution? As chordates innovated a tight junction in most of their tissues, and only maintained a SJ-like junction in the paranode of myelinated axons [113], SJ proteins may have evolved new functions, but may also have retained some of the non-occluding functions described here. Examining the expression and function of the true orthologs of SJ proteins in mice and fish have the potential to address this question and further our understanding of this fascinating collection of proteins.

## Figures and Tables

**Table 1:** Protein composition of the SJ.

Protein	Core/Resident/Accessory	Protein Domains	References
ATPα	Core	P-type cationic transporter	[21,38]
Contactin	Core	C-type lectin-like, Ig, FN3	[26,39]
Coracle	Core	FERM	[18,27]
Crimpled	Core	Ly6	[34,40]
Kune-kune	Core	PMP-22/claudin	[25]
Lachesin	Core	Ig	[41]
Macroglobulin complement-related	Core	LDL Rec A, α2 macroglobulin	[22,23]
Megatrachea (Pickel)	Core	PMP-22/claudin	[42]
Nervana 2	Core	Na/K ATPase β subunit	[38]
Neurexin-IV	Core	FA58C, LamG, EGF-like	[20,39]
Neuroglian	Core	Ig, FN3	[21]
Pasiflora 1	Core	None (4 TM)	[43]
Pasiflora 2	Core	None (4 TM)	[43]
Sinuous	Core	PMP-22/claudin	[24]
Transferrin 2 (Melanotransferrin)	Core	Transferrin-like	[37]
Varicose	Core	PDZ, SH3, GUK	[44,45]
Würmchen	Core	None	[46]
Bark Beetle/Anakonda	tSJ	SRCR, Parallel beta-helix	[11,12]
Gliotactin	tSJ	Carboxylesterase	[10]
M6	tSJ	Myelin PLP	[13]
Discs Large	resident	PDZ, SH3, GUK	[29]
Fasciclin III	resident	Ig C2-set	[47]
Lethal (2) giant larvae	partial resident	WD40 repeats	[48]
Scribble	resident	Leucine-rich, PDZ	[30]
Boudin	accessory	Ly6	[33]
Clathrin heavy chain	accessory	Clathrin heavy chain repeat	[37,49]
Coiled	accessory	Ly6	[32,34,35]
Crooked	accessory	Ly6	[34]
Rab 5	accessory	Small GTPase	[37]
Rab 11	accessory	Small GTPase	[37]
Shibire	accessory	Dynamin GTPase and effector domains, PH	[37,49]
Wunen 1	accessory	PA phosphatase type 2	[36]
Wunen 2	accessory	PA phosphatase type 2	[36]
